# Adhesive forces and surface properties of cold gas plasma treated UHMWPE

**DOI:** 10.1016/j.colsurfa.2014.03.052

**Published:** 2014-10-20

**Authors:** Emily Callard Preedy, Emmanuel Brousseau, Sam L. Evans, Stefano Perni, Polina Prokopovich

**Affiliations:** a Cardiff School of Pharmacy and Pharmaceutical Science, Cardiff University, Redwood Building, King Edward VII Avenue, Cardiff CF10 3NB, Wales, UK; b Cardiff School of Engineering, Cardiff University, Queen's Buildings, The Parade, Cardiff CF24 3AA, Wales, UK; c Department of Biological Engineering, Massachusetts Institute of Technology, 77 Massachusetts Avenue, NE47-377, Cambridge, MA 02139, USA

**Keywords:** A.C., alternative current, AFM, atomic force microscopy, CAP, cold atmospheric plasma, ECM, extracellular matrix, PBS, phosphate buffer solution, PCTFE, polychlorofluoroethylene, slm, standard litre per minute, sccm, standard cubic centimetre per minute, TJA, total joint arthroplasty, TJR, total joint replacement, UHMWPE, ultra-high molecular weight polyethylene, XLPE, highly cross-linked polyethylene, UHMWPE, Cold atmospheric plasma-treatment, Adhesion forces, Surface topography, Material modification, AFM

## Abstract

Cold atmospheric plasma (CAP) treatment was used on ultra-high molecular weight polyethylene (UHMWPE), a common articulating counter material employed in hip and knee replacements. UHMWPE is a biocompatible polymer with low friction coefficient, yet does not have robust wear characteristics. CAP effectively cross-links the polymer chains of the UHMWPE improving wear performance (Perni et al., Acta Biomater. 8(3) (2012) 1357).

In this work, interactions between CAP treated UHMWPE and spherical borosilicate sphere (representing model material for bone) were considered employing AFM technique. Adhesive forces increased, in the presence of PBS, after treatment with helium and helium/oxygen cold gas plasmas. Furthermore, a more hydrophilic surface of UHMWPE was observed after both treatments, determined through a reduction of up to a third in the contact angles of water. On the other hand, the asperity density also decreased by half, yet the asperity height had a three-fold decrease. This work shows that CAP treatment can be a very effective technique at enhancing the adhesion between bone and UHMWPE implant material as aided by the increased adhesion forces. Moreover, the hydrophilicity of the CAP treated UHMWPE can lead to proteins and cells adhesion to the surface of the implant stimulating osseointegration process.

## Highlights

• He and He/O_2_ cold gas plasma (CAP) were used to surface modify UHMWPE.
• CAP reduced the UHMWPE asperity density and their height through plasma etching.
• CAP improved hydrophilicity of UHMWPE.
• Adhesion between a borosilicate sphere and CAP treated UHMWPE were measured.
• CAP modified UHMWPE had higher adhesion forces.

## Graphical abstract

**Figure:**
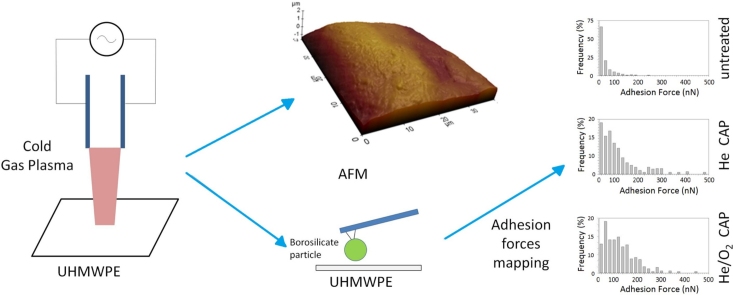

## 1 Introduction

Osteoarthritis is also known as “degenerative joint disease” and occurs as a result of loss in articular cartilage which lines the bone of synovial joints [1]. Articular cartilage minimises stress on subchondral bone and provides low friction surfaces [2], [3] playing an essential role in these lubricating junctions. Furthermore, it is a resilient tissue, demonstrating features of durability [3] through compression and shear. However, once the cartilage fractures, it has limited or no ability to heal. These injures are often caused by mechanical twisting and direct impact/loading, including: direct injury to the articular cartilage (e.g. osteochondral fractures); abnormal mechanical stress on the joint (e.g. in poor joint alignment),impaired subchondral bone support and blood supply (e.g. avascular necrosis). These events can alter the composition, along with the structure, and mechanical properties of the cartilage impairing its ability to perform the required functions [3], [4].

In many cases, if the medical management i.e. drug intervention of the joints has failed then many patients are recommended for total joint arthroplasty (TJA) [5]. It is well accepted, since its development in the 1960s [6], that total hip and total knee replacement surgery is a reliable method to relieve pain and return lower limb function, generally to improve the quality of life for the patient. Ultra high molecular weight polyethylene (UHMWPE) has been commonly used for over four decades [7], [8] as an articulating counter surface for TJA. For example, UHMWPE is used in spine disk replacement [9], as a concave bearing material in the acetabular cup in the hip, and as the tibial tray in the knee; with the opposite bearing surface traditionally made of hard but very smooth ceramic (alumina, zirconia, hydroxyapatite/calcium phosphate) or metal (stainless steel, titanium and alloys, cobalt and alloy materials. Other polymers (polyethylene, polypropylene, polyurethane, poly-methyl methacrylate) [10] in combination with metal/alloys (nickel–titanium; cobalt–chromium) are also used as they have good mechanical and surface properties when employed together [11]. However, the true success of UHMWPE lies in the fact that it is biocompatible [11], with good low friction properties, chemical inertness, high impact strength and sufficient mechanical performance [12], [13], [14].

Regardless of the hype associated with UHMWPE, it does not display robust wear performance on dynamic load [15] as the energy builds up and accumulates forming cracks [15]; therefore, decreasing the longevity of the device for TJA [13]. Research has suggested that some wear particles, of polyethylene in particular, are the result of the sterilisation treatment pre-surgery which introduces free radicals [13], [16] and is often achieved through gamma radiation [15]. Certain techniques have already been used as an attempt at improving the wear performance of UHMWPE, for example: gamma or electron beam irradiation with thermal stabilisation [13], [17]; ion bombardment [18]; proton radiation [14]; argon plasma surface modification [19] and addition of Vitamin E to the polymer [20]. Irradiation of the polymer was introduced to overcome the initial problem of fragility; gamma radiation effectively produced free carbon radicals on the backbone chain of the polymer which caused cross-linking (desirable), chain scission, and oxidation (undesirable) of the polymer [15], [16]. Crosslinking increases the wear resistance, abrasion resistance, and even improve the thermal stability [14], [15]; on the other hand oxidation has a negative effect by decreasing the properties listed as well as lowering the molecular weight and chain length governing the fragility [15].

Surface processing with gas plasma effectively alters the surface chemistry of the material by the bombardment of ions, electrons UV radiation and other chemically reactive species (radicals) present in the plasma plume [13]. Developments in gas plasma technology allow generating gas plasmas at room temperature without the need for vacuum, these have been called “cold atmospheric plasmas” (CAP) [13], [21]; this is a cheaper alternative than other plasma techniques as it is performed in air and without a vacuum chamber; it also aids as a sterilising technique. Cold gas plasma technology has been successfully employed to enhance the wear performance of UHMWPE [13]; untreated UHMWPE had a wear factor of 2.7 × 10^−7^ mm^3^/N/m, yet after just 7 min of treatment with the plasmas, it had a wear factor of almost half the untreated material at 1.4 × 10^−7^ mm^3^/N/m; further benefits were also seen after XRD diffraction patterns demonstrated that the treatments did not affect the crystallinity of the UHMWPE, therefore maintaining its integrity and in retaining cytocompatible properties of untreated UHMWPE [13]. Although, treatment improved the wear performance of the UHMWPE, no evidence of the CAP on the adhesive properties outcome of the material has been studied which inherently cause wear particles. On the other hand, adhesion plays an essential role when applied to osseointegration; which is the process of bone adhering to the implanted device to improve function and overall health of the patient [22]. Therefore, in this study these characteristics of the new materials (CAP modified UHMWPE) were investigated by delving further into the role of adhesive force interactions focusing on the comparison of the untreated and CAP treated UHMWPE and by presenting comprehensive surface topography analysis along with adhesive force mapping, using atomic force microscopy (AFM); the impact of the treatment on the UHMWPE properties was also characterised by contact angle measurements and surface energy parameters determination.

## 2 Materials and methods

### 2.1 Polymers

Selections of UHMWPE (GUR 1020, Hoechst, Germany) were employed in this study.

### 2.2 Cold gas plasma treatment

The CAP device was made of two electrodes: a capillary powered electrode, situated within a quartz tube of 1.5 mm inner diameter; and a downstream ring electrode, this is wrapped around the outside of the quartz tube near a nozzle, allowing for the axial separation of the electrodes producing an axially directed electrical field upon an external voltage.

Helium plasmas were generated allowing 5 slm (standard litre per minute) of 99.99% helium to flow through the capillary electrode [13], helium/oxygen plasmas were generated mixing 10 sccm (standard cubic centimetre per minute) of oxygen to the helium gas flow before entering the CAP device.

Power was supplied by an alternative current (A.C.) with a peak voltage of 8 kV and an excitation frequency of 20 kHz [13]. Due to the alignment of the electrodes and hence the electrical field, then the gas flow is also in an axial direction and is sometimes known as linear field devices [13], [23], [24]. The resulting applied voltage governs the breakdown of the gas to produce an electrical discharge inside the quartz tube, which appears as a light emitting plume or plasma jet from the quartz [23].

### 2.3 Surface analysis

Atomic Force Microscope (AFM) (XE-100 Advanced Scanning Probe Microscope (Park Systems, Korea) was used for surface topography analysis, as well as for adhesion force measurements.

#### 2.3.1 Topography

In order to image all three samples (untreated UHMWPE, Treated UHMWPE-Helium, Helium and Oxygen mix), a contact rectangular tip, CSG30 (NT-MDT, The Netherlands), with reflective Au side, was used with a spring constant of 3.3 N/m, a tip height of 14 μm and a tip curvature of radius of 10 nm. This probe was calibrated using the Sader method [25].

The scan parameters used were as follows: scan size of 40 × 40 μm; resolution at 1024 × 1024; scan rate was maintained between 0.8 and 1.0 Hz and an applied load of 21.34 nN. Three independent samples for each of the material used were scanned and around 10 images for each sample were taken.

Asperities were located using an in-house written FORTRAN code under the following conditions: a point on the surface is an asperity if the 8 bordering points (in *x* and *y* directions) are of lower height and the *z*-coordinates bordering these are further away as well as lower in height [26]. Once located, the asperity height, density and the radius of curvature were determined using the in-house built FORTRAN code, as described in detail in other work [26].

Furthermore, the parameter *R*_*f*_ of the Wenzel equation was calculated as described previously [26].

(1) $$R_{f}=\frac{A_{SL}}{A_{F}}$$

where *A*_*SL*_ is the actual solid interface and *A*_*F*_ is its projection on a flat plane.

#### 2.3.2 Surface energy

The surface energy components for each material were determined by contact angle measurements of water (*θ*_*w*_), ethylene glycol (*θ*_*et*_) and hexadecane (*θ*_*h*_) as described in [27]. A 5 μl, drop of each liquid, was gently placed onto the UHMWPE samples and imaged using a digital camera; 10 replicates were performed on each sample. The contact angle of both the right and left side of the liquid drop were measured using ImageJ software (NIH, USA). The mean values of the contact angles measured were used along with the surface energy parameters: Lifshitz–Van der Waals interactions (*γ*^LW^), Lewis acid–base interactions *γ*^AB^, the electron-acceptor (*γ*^+^) and the electron-donor (*γ*^−^) molecular interaction through the following equation:

(2) $$\gamma_{L}(1-\cos\theta )=2\left(\sqrt{\gamma_{S}^{LW}\gamma_{L}^{LW}+\sqrt{\gamma_{S}^{+}\gamma_{L}^{-}}+\sqrt{\gamma_{S}^{-}\gamma_{L}^{+}}}\right)\text{.}$$

#### 2.3.3 Adhesive force measurements

All adhesive force measurements were conducted in an open liquid cell which was made of polychlorofluoroethylene, PCTFE (Park Systems, Korea), a homopolymer with high compressive strength and low deformation under load; PBS was used as aqueous phase. A rectangular silicone cantilever with a borosilicate colloid sphere, 20 μm in diameter, attached (Novoscan, USA) was used with a spring constant 14 N/m calibrated using Craig and Neto's in situ calibration of colloidal probe cantilevers [25], [28], [29], with Au surface (no reflex).

In order to gain comprehensive data for the adhesive interactions for the given samples, the surface mapping feature of the AFM was employed. Using a 40 × 40 μm scan size, 144 force curves were obtained on each UHMWPE samples, as three different samples were measured for each untreated and CAP treated UHMWPE, a total of 1296 force curves were analysed for every material for this work.

### 2.4 Statistical analysis

Asperity heights, curvature radii and forces of adhesion distribution were tested for Gaussian behaviour using the chi-square test (*χ*^2^ test). The effect of the cold gas plasma treatment on the asperity heights distribution was investigated through the one-way ANOVA [30], to determine any significant differences between the means as the samples are independent of each other [31]; followed by Bonferroni *post-hoc* correction [32] test (*p* < 0.05) which enables adjustments by overcompensating for the multiple comparisons, and is based on the multiplication of each significance levels from the least significant difference by the number of tests performed [33]. The differences in the asperity curvature radii and adhesion forces were analysed with Kruskal–Wallis test, used for independent data that do not follow normal distribution and compares the medians of the samples; followed *post hoc* with a Dunn's test, a multiple comparison correction test similar to the Bonferroni but analyses the variance when the number of comparisons is not large [34], [35].

## 3 Results

### 3.1 Surface topography

**Fig. 1:**
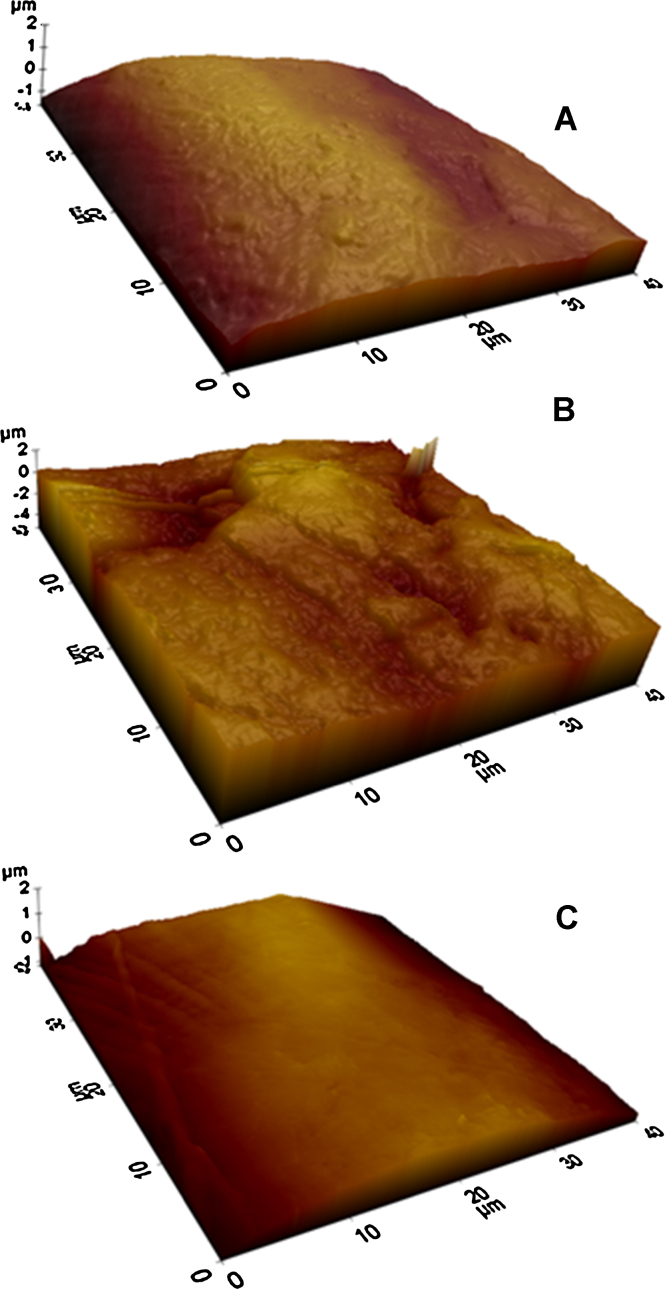
AFM 3D images of UHMWPE pre- (A) and post-CPA treatment with helium (B) and helium oxygen mix (C).

**Fig. 2:**
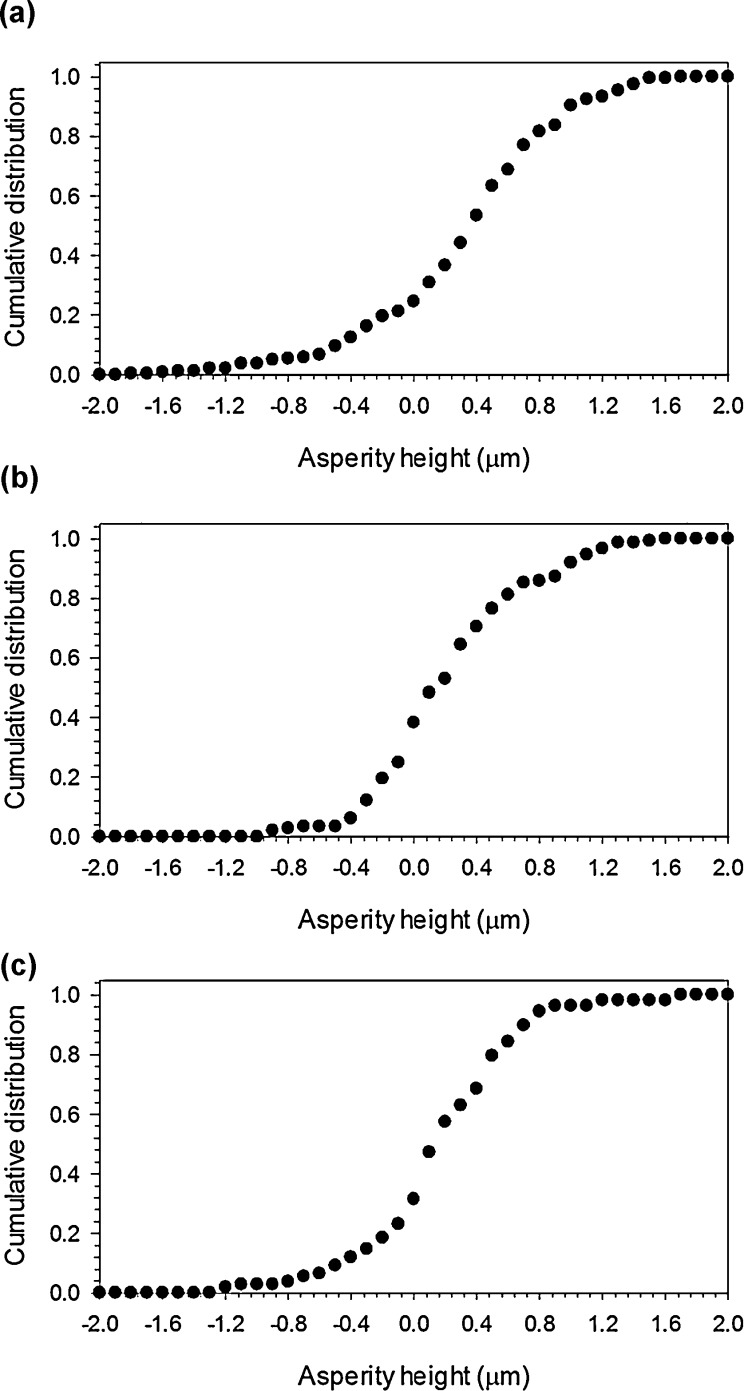
Cumulative frequency distribution data of the asperity heights untreated (A) and post-CPA treatment with Helium (B) and helium/oxygen mix (C).

**Fig. 3:**
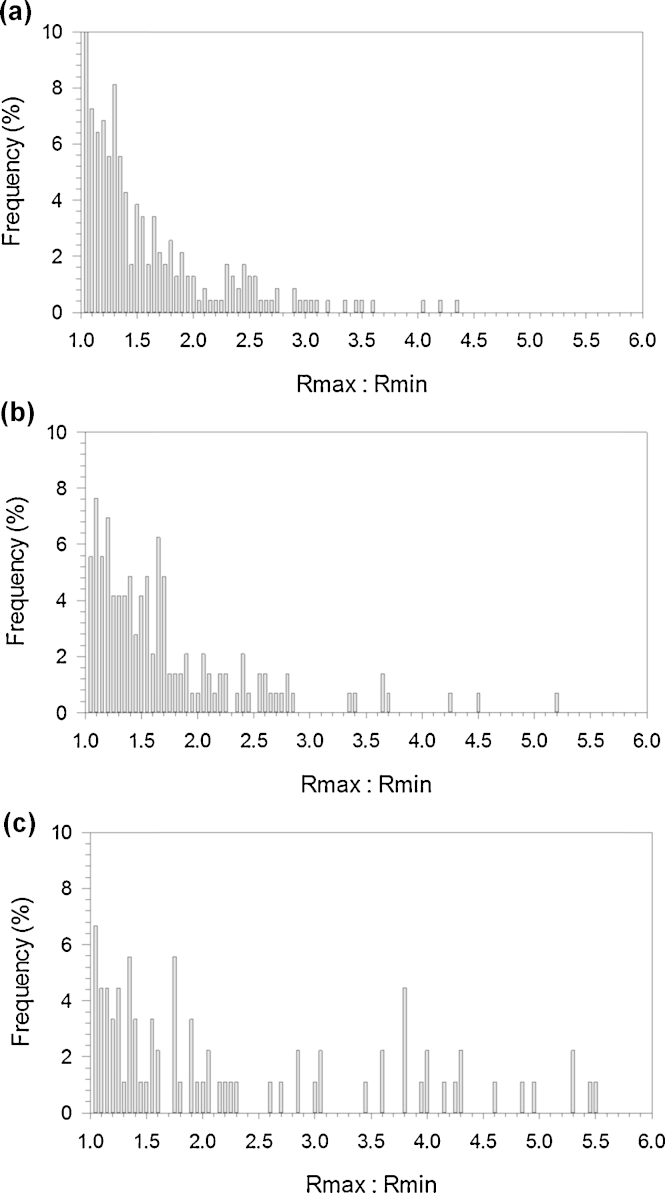
Distributions for the ratios of curvature of radii in orthogonal directions (*R*_max_/*R*_min_) for all samples of UHMWPE: untreated (A); helium treated (B) and helium/oxygen mix treated (C).

Analysis of the 40 × 40 μm scanned areas of both untreated and CAP treated UHMWPE helium and helium/oxygen mixture (Fig. 1) estimated that the average asperity density for untreated UHMWPE was 15.2 × 10^10^ asp/m^2^, almost twice the value after CAP treatment of both Helium and the Helium and Oxygen mix samples, 9.3 × 10^10^ asp/m^2^ and 6.7 × 10^10^ asp/m^2^, respectively (Table 1). Another parameter which attributes to the surface roughness of the samples is the asperity height; all samples of UHMWPE both treated and untreated had asperity heights normally distributed (*p* < 0.01) and the cumulative distributions of this parameters are shown in Fig. 2. It is clear from the results that a three-fold reduction of asperity height has occurred post-CAP treatment of the samples (*p* < 0.01) with no significant difference between the CAP-treated samples (*p* > 0.05); as the untreated UHMWPE had an average asperity height of 652 nm yet the Helium treated UHMWPE had an average asperity height of 223 nm, while the Helium and Oxygen mixture treated samples average asperity height reduced to 180 nm. Changes in the *R*_*f*_ factor were similar to those of asperity heights; CAP treated samples exhibited *R*_*f*_ of about 1.08 regardless of the gas used; whilst untreated UHMWPE has a *R*_*f*_ of 1.18. A further surface topographical aspect which has been investigated was the radii of curvature of asperities. The distributions of the ratios of the asperity radii in the orthogonal *x* and *y* directions (larger radius between *R*_*x*_ and *R*_*y*_ divided by the smaller) were investigated to determine the hemisphericity of the asperities. Untreated UHMWPE surface had asperities with a hemispherical shape as the majority of ratios was in the range 1–2; similarly for helium and helium/oxygen CAP treated UHMWPE (Fig. 3). When considering the percentile results (Table 1), it emerged that in all cases the curvature radii were not normally distributed (*p* > 0.05). Moreover, the He/O_2_ cold gas plasma treatment did not result in a statistical difference compared to untreated samples, whereas the Helium cold gas plasma caused a reduction of the curvature asperity radii.

**Table 1:** Surface topography analysis data recovered from each of the three UHMWPE samples.

UHMWPE Samples	Asperity density (Asp/m^2^)	Average Asperity height (nm)	Percentile of Radius of curvature in *x* and *y* direction		
			25th (μm)	50th (μm)	75th (μm)
Untreated	15.2 ± 0.3 E + 10	652 ± 25	1.55 ± 0.11	2.33 ± 0.05	3.66 ± 0.05
He	9.3 ± 1.1 E + 10	223 ± 47	0.64 ± 0.26	1.12 ± 0.13	2.37 ± 0.84
He/O_2_	6.7 ± 0.9 E + 10	180 ± 53	1.13 ± 0.42	2.46 ± 0.54	3.49 ± 0.76

### 3.2 Contact angles and surface energy

All surface energy parameters, including the contact angles, are given in Table 2. The contact angle measurements display great changes as there is a decrease in contact angle for both CAP treated samples compared to the untreated UHMWPE. For example, contact area of water drop, *θ*_*w*_, was found to be 70.4̊ for untreated UHMWPE, but for helium and helium/oxygen CAP treated UHMWPE the angle was measured at 46.1̊ and 58.0̊, respectively. This decreasing trend was also found for the contact angles for ethylene glycol, *θ*_*et*_, for example, *θ*_*et*_: 51.2̊ for untreated UHMWPE; 35.3̊ helium CAP treated UHMWPE, however this decrease was not noticed for helium/oxygen CAP treated UHMWPE. On the other hand the surface energy data clearly demonstrates that there is little difference of the electron-donor and electron-acceptor parameters (*γ*^AB^), with results varying by a few mJ/m^2^: 7.89, 8.38 and 6.54 mJ/m^2^ for untreated UHMWPE and CAP-treated UHMWPE with helium and helium/oxygen mixture, respectively. As the dispersive surface free energies (*γ*^LW^) are also calculated also had little differences between the samples, for untreated UHMWPE, 25.58 mJ/m^2^; 26.61 mJ/m^2^ for the helium treated sample and for the helium and oxygen mix sample that was tested was 30.23 mJ/m^2^. Therefore due to the small difference, the overall total of the surface free energies for the tested samples not surprisingly had little variation of 33.47 mJ/m^2^; 34.99 mJ/m^2^; and 36.77 mJ/m^2^ for untreated UHMWPE, and CAP-treated UHMWPE with helium, and helium oxygen mix, respectively.

**Table 2:** Contact angles of water (*θ*_*w*_), ethylene glycol (*θ*_*et*_), hexadecane (*θ*_*h*_) and surface energy parameters of UHMWPE samples.

	$\theta_{w}$	*θ*_*et*_	*θ*_*h*_	*γ*^LW^ (mJ/m^2^)	*γ*^+^ (mJ/m^2^)	*γ*^−^ (mJ/m^2^)	*γ*^AB^ (mJ/m^2^)	*γ*^tot^ (mJ/m^2^)
Untreated	70.4 ± 1.5	51.2 ± 1.9	19.7 ± 2.4	25.58	0.92	16.93	7.89	33.47
He	46.1 ± 2.5	35.3 ± 2.6	12.4 ± 1.7	26.61	1.04	17.00	8.38	34.99
He/O_2_	58.0 ± 3.6	50.2 ± 3.8	15.0 ± 3.1	30.23	0.33	32.73	6.54	36.77

### 3.3 Adhesion force measurements

**Fig. 4:**
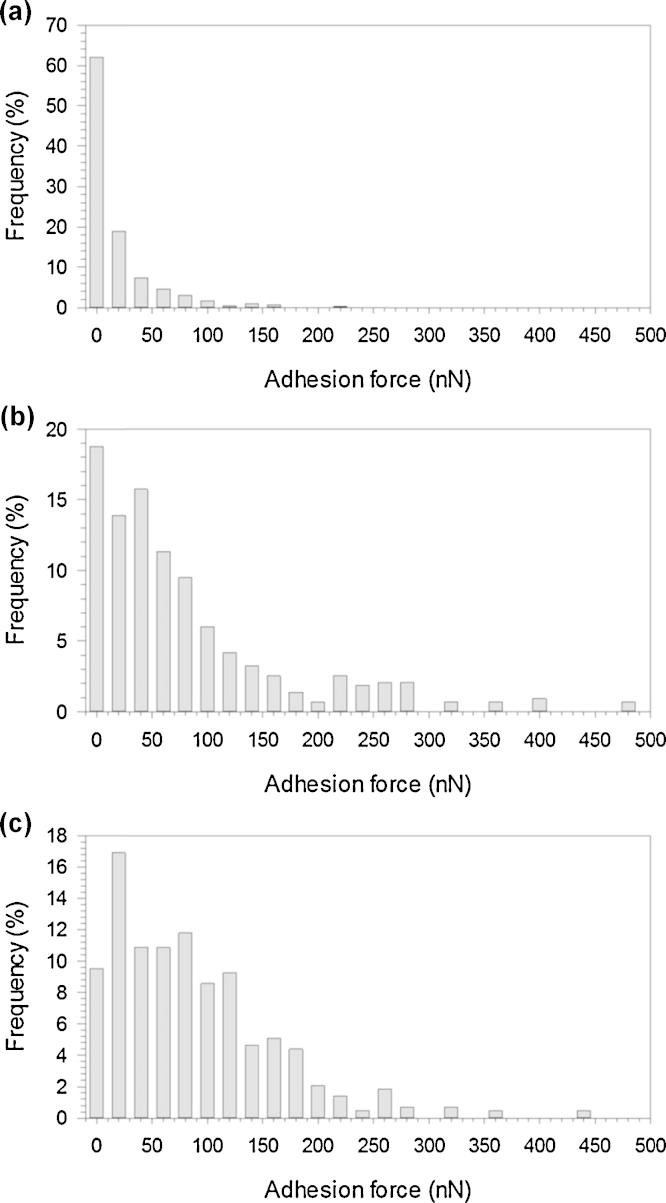
Frequency distribution of the adhesion forces for all tested samples of UHMWPE; (a) untreated; (b) helium treated and (c) helium/oxygen mix.

Adhesion force measurements results exhibited a non-normal distribution (*p* > 0.05) (Fig. 4). The resulting adhesion measurements for untreated UHMWPE in PBS had a median adhesion force measurement of around 10 nN, whereas both treated samples had a median adhesion force about 50 nN. The Kruskal–Wallis test applied to the adhesion forces data revealed that CAP treated samples did not show statistically significant differences (*p* > 0.05) whilst the untreated UHMWPE had adhesion forces statistically different from the other two samples (*p* < 0.01).

## 4 Discussion

Irregularities on the material surface are features exhibited by all solid materials; these are denoted “asperities”. For decades adhesion has been of interest in research [36], [37], [38], [39] and is a complex phenomenon applied to a vast range of activities from walking to bacterial attachment [38]. Generally, there are many aspects which govern the adhesion process, for example, the surface topography may influence the extent of adhesion due to variations in the real area of contact [26].

Moreover, the physicochemical properties associated with the surface of UHMWPE are strongly influenced by the chemical and functional groups of the polymer. In this case, polyethylene polymer is a long chain consisting of the monomer ethylene ((C_2_H_4_)_*n*_) [40], variations exist in the density and amount of branching. However, as discussed, UHMWPE has a disadvantage in regards to wear leading to aseptic loosening [8], [12], [40]. It has been highlighted that highly cross-linked polyethylene (XLPE) [40] has a higher density and usually achieved through costly process such as thermal treatment and irradiation; and has been noted that these procedures introduce another problem of oxidation [15], [16] causing long-term damage and failure of the device. Therefore, the CAP treatment has the advantages of these techniques through introducing free radicals such as nitrogen from the surrounding environment, promoting further cross-linking across multiple polymer chains, effectively increasing the density and strength of the polymer [40], without the negative outcomes often associated with thermal treatments. We have previously demonstrated, using RAMAN spectroscopy, that nitrogen groups are generated on UHMWPE as result of CAP treatment [13]. Furthermore, we have proved here that these groups have a clear effect on the surface properties of the samples post-CAP treatment increasing the hydrophilicity of the material. Another reason for the decrease in contact angle after CAP treatment can be the increase of roughness parameter according to the Wenzel relation (non-composite wetting state):

(3) $$\cos\vartheta =R_{f}\cos\vartheta_{0}$$

where ϑ is the contact angle on a rough surface, ϑ_0_ is the contact angle on a smooth surface and *R*_*f*_ is the roughness factor defined in Eq. 1.

However this does not seem to be the case in this work as the variation of *R*_*f*_ values associated with CAP treatment was not pronounced and could not account for the reduction of contact angles of water. Therefore, changing in surface chemistry is the prevailing reason for lowing contact angle of water.

As was observed in this work, the changes in the surface topography are inherent of plasma etching which explains the decreased asperity density by almost half that of the untreated UHMWPE compared to both CAP treated UHMWPE, as well as the reduction in height. The plasma bombards the surface with highly charged ions/radicals that etch away parts of the surface, some of these parts may embed to the surface through chemical reactions therefore disrupting the topography; such as shaving the height of asperities, thereby reducing the number of asperities per given area. Also, plasma etching may have an effect on the curvature of radii in a similar manner as many engineering applications such as machining processes which often produce asperities that do not exhibit normal distribution [26], [41]. The variations in height and shape of asperity play an essential role in the adhesion phenomena and influence the resulting adhesion force measurements along for a closer apposition of bone to the implanted device [42].

It is evident that the mechanical and physicochemical interactions between the implant and lubricant is a necessity [43], especially when considering osseointegration [44] and the formation of molecular layers to the implant to minimise friction and potential wear. Osseointegration has been defined as direct structural and functional connection between ordered living bone and implant [22]. For example in TJR surgery, water forms a thin layer which facilitates the adsorption of proteins onto the surface of the implanted device; this is commonly known as the conditioning film which initiates cellular adhesion [22]. Normally by day 5 post surgery, new bone formation is essential [22]; this process is governed by the surface properties of the material and the adsorption rate of osteoblast cells on the surface. The proliferation of osteoblasts leads to acceptance of the implant, otherwise rejection occurs [44]. It has also been pointed out [45] that the biocompatibility of the implant, as well as the topography, chemistry and surface energy can influence the cell behaviour and, therefore, attachment. Moreover, these studies indicate that an increased adhesion and hydrophilicity of the polymers surface lead to improved osseointegration [44], [46].

## 5 Conclusion

This work aimed at comparing the effects of CAP-treatment with helium and helium/oxygen cold gas plasmas on medical grade UHMWPE surface properties and forces of adhesion. Results showed that CAP treatment decreased the asperity density of both treated samples by half that of the untreated UHMWPE; the treatment also reduced the height of these asperities due to plasma etching. However, the CAP-treatment did not affect the overall surface energy of the tested samples as there was little difference in the surface energy parameters, although a decrease in contact angle of water was noticed in the treated UHMWPE samples, thereby improving the hydrophilicity. After CAP treatment, higher adhesion forces were measured between UHMWPE samples and a boro-silicate particle.

These results show that CAP-treated UHMWPE has advantageous characteristics associated with successful prostheses; not only better wear performance as previously proven, but also increased adhesion which is essential for osseointegration.
